# Versatile Imidazole Scaffold with Potent Activity against Multiple Apicomplexan Parasites

**DOI:** 10.1021/acsinfecdis.5c00049

**Published:** 2025-05-08

**Authors:** Monique Khim, Jemma Montgomery, Mariana Laureano De Souza, Melvin Delvillar, Lyssa J. Weible, Mayuri Prabakaran, Matthew A. Hulverson, Tyler Eck, Rammohan Y. Bheemanabonia, P. Holland Alday, David P. Rotella, J. Stone Doggett, Bart L. Staker, Kayode K. Ojo, Purnima Bhanot

**Affiliations:** † Seattle Structural Genomics Center for Infectious Disease, Seattle, Washington 98109, United States; ‡ Center for Global Infectious Disease Research 145793 Seattle Children’s Research Institute, Seattle, Washington 98109, United States; § Divisions of Infectious Diseases and Research VA Portland Healthcare System, Portland, Oregon 97239, United States; ∥ Department of Microbiology, Biochemistry and Molecular Genetics, 12286 Rutgers New Jersey Medical School, Newark, New Jersey 07103, United States; ⊥ Department of Medicine, Division of Allergy and Infectious Diseases, Center for Emerging and Reemerging Infectious Diseases 12353 University of Washington, Seattle, Washington 98109, United States; # Department of Chemistry and Biochemistry and Sokol Institute of Pharmaceutical Life Sciences 8087 Montclair State University, Montclair, New Jersey 07043, United States; ¶ Division of Infectious Diseases,Oregon Health & Science University School of Medicine, Portland, Oregon 97239, United States; ∇ Department of Global Health University of Washington, Seattle, Washington 98195, United States

**Keywords:** kinase, drugs, apicomplexa, Plasmodium, Toxoplasma

## Abstract

Malaria, toxoplasmosis, and cryptosporidiosis are caused by apicomplexan parasites spp., , and , respectively, and pose major health challenges. Their therapies are inadequate, ineffective or threatened by drug resistance. The development of novel drugs against them requires innovative and resource-efficient strategies. We exploited the kinome conservation of these parasites to determine the cellular targets and effects of two inhibitors in and . The imidazoles, (*R*)-RY-1-165 and (*R*)-RY-1-185, were developed to target the cGMP dependent protein kinase of (PfPKG), orthologs of which are present in and . Using structural and modeling approaches we determined that the molecules bind stereospecifically and interact with PfPKG in a manner unique among described inhibitors. We used enzymatic assays and mutant expressing PfPKG with a substituted “gatekeeper” residue to determine that cellular activity of the molecules is mediated through targets additional to PfPKG. These likely include calcium dependent protein kinase 1 and 4 (PfCDPK-1, -4), kinases that, like PfPKG, have small amino acids at the “gatekeeper” position. The molecules are active against and , with tachyzoites being particularly sensitive. Using mutant parasites, enzyme assays and modeling studies we demonstrate that targets in include TgPKG, TgCDPK1, TgCDPK4 and the mitogen activated kinase-like 1 (MAPKL-1). Our results suggest that this scaffold holds promise for the development of new toxoplasmosis drugs.

## Graphical abstract

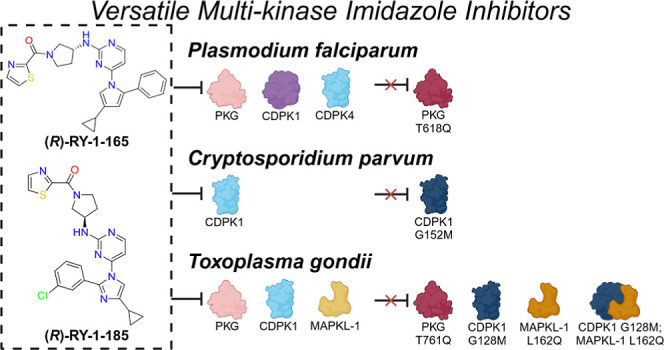

Malaria, toxoplasmosis, and cryptosporidiosis are caused by apicomplexan parasites, spp. (five species), , spp. (two species), respectively. They are the major parasitic diseases in humans, with significant mortality and morbidity worldwide.^1–3^ Malaria caused an estimated 608,000 deaths in 2022 alone. There were an additional 249 million cases spread across 85 countries.^1^ Nearly 75% of all malaria deaths were of children under five-years in sub-Saharan Africa. Resistance to frontline treatments, based on artemisinin-containing drug combinations, is common in Southeast Asia and has emerged in several African countries.^4^ Toxoplasmosis in immunocompromised patients can be deadly and in pregnant women can cause stillbirth, miscarriage, and birth defects.^5^ In addition, two billion people worldwide are chronically infected with .^5^ These latent infections can be reactivated upon weakening or suppression of the immune system. Current therapies for toxoplasmosis are ineffective against latent forms of that cause chronic infection. Furthermore, drugs used to treat acute toxoplasmosis induce serious side-effects in a substantial number of patients.^6^ Cryptosporidiosis, caused by and , is among the leading causes of pediatric diarrhea in under-resourced countries.^3,7^ The global prevalence of cryptosporidium infection is estimated to be 7.6%.^8^ While the infection is self-limiting in immunocompetent and well-nourished adults, infants and immunocompromised adults experience moderate to severe chronic diarrhea that can result in malnourishment, stunted growth and death. Standard therapy for cryptosporidiosis relies on a single FDA-approved drug and this has shown little-to-no efficacy in populations that are most at risk.^9^

Given that current therapies against malaria, toxoplasmosis, and cryptosporidiosis are threatened by drug resistance or are insufficient or ineffective in at-risk populations, there is a clear need for new drugs against these diseases. Since the three parasites are intracellular protozoans of the phylum, there are many similarities in their biology and a high degree of evolutionary conservation in their genomes. These common features enable parasite-hopping to be a resource-efficient route to early stage drug discovery against individual parasites. The conserved kinomes of these parasites is a rich source of such opportunities.

Selective inhibition of parasite kinases over their human counterparts is often achievable by exploiting a common and key difference between their ATP-binding pockets. The ATP-binding pockets of most human kinases carry large amino acids, such as a Met, Phe, Tyr, Leu, and Gln, at a site termed the “gatekeeper” that lies deep in the ATP-binding pocket,^10^ while several essential parasite kinases have smaller amino acids such as Thr, Ser, Cys, Gly, and Ala. This increases the volume available in the parasite kinase to fit inhibitory molecules that spare their human homologue, leading to significant pathogen-selective activity. The rational design of such compounds is also facilitated by the availability of high-resolution structures for one or more parasite kinase homologues.^11^

The strategy of leveraging small gatekeeper residues to provide target selectivity has been successfully deployed to target cGMP dependent protein kinases (PKG) of and , and calcium-dependent protein kinases (CDPKs) of , and .^12–14^ The ‘gatekeeper’ of PKG (PfPKG), PKG (TgPKG) and or PKG (Cp/ChPKG) is a Thr, which has a shorter side chain compared to the Met which occupies the gatekeeper position in human PKG (hPKG).^15^ CDPK4 has a Ser gatekeeper residue. Its closest homologues in (TgCDPK1) and (CpCDPK1) have a Gly at the equivalent position.^16^ PfCDPK1 contains a Thr gatekeeper residue.

In addition to small “gatekeeper” residues, another attribute in common among these homologous kinases is their indispensability in one or more stages of the respective parasites. PfCDPK1 and PfCDPK4 are required for the production of gametes and oocysts in the mosquito.^17,18^ Hence, they are essential for parasite transmission from human to mosquito and development within the vector. TgCDPK1 is required for tachyzoite motility, invasion and egress and hence proliferation.^19^ CpCDPK1 is essential for survival.^20^ Consequently, TgCDPK1 and CpCDPK1 are the targets of active drug discovery efforts.^21,22^ PfPKG is essential for multiple steps of life cycle in the human host and the mosquito vector.^23,24^ There is crosstalk between kinase pathways and a degree of redundancy in their action.^25^ Simultaneous pharmacological inhibition of two or more of these kinases may be more efficacious in controlling parasite growth than highly specific inhibition, provided that high selectivity for the parasite is maintained.

Of the kinases mentioned above, PfPKG is the most thoroughly validated target in . It is essential for the parasite’s erythrocytic cycle since its genetic depletion or chemical inhibition blocks merozoite invasion and egress from infected erythrocytes.^24^ Work in the rodent-infective species, demonstrated its role in sporozoite invasion of and exit from hepatocytes.^26–29^ In addition, it is essential for gametogenesis in the mosquito vector.^13^ In accordance with these functions, pharmacological inhibition of PfPKG shows promise in blocking the asexual erythrocytic cycle of in mice engrafted with human erythrocytes^30^ and preventing infection by sporozoites in mouse liver.^27^ An additional feature that makes PfPKG an attractive drug target is that, in laboratory-evolved experiments, either does not develop resistance to its inhibitors or does so minimally.^24^ These characteristics have spurred development of PfPKG inhibitors for the treatment and prevention of infection.^23,24^ Similar to the enzyme, TgPKG is essential for tachyzoite lytic cycle,^12^ hence essential for parasite growth and survival. CpPKG is also essential in as it is required for egress of merozoites from epithelial cells.^31^

We previously described two imidazole compounds, RY-1-165 and RY-1-185 (14a and 14b in ref 32) with potency against PfPKG, and activity against asexual stages and sporozoites. In this study, we used structural and computational approaches to explore their polypharmacology and predict additional targets. We took a parasite-hopping opportunity by determining potency in vitro against enzyme targets from and , and testing activity against the two parasites. We find that is highly sensitive to RY-1-165 and RY-1-185 relative to and . Our findings support further investigation of the scaffold for novel drugs for toxoplasmosis.

## Results

### (*R*)-RY-1-165 Contains a Unique Biaryl Moiety, Which Binds Stereospecifically and Displaces the c-Terminal Tail

We recently reported development of PfPKG inhibitors based on an imidazole core and identified structure–activity relationship (SAR) models of inhibition.^32^ The R-enantiomer of RY-1-165 is a potent inhibitor of PfPKG while the S-enantiomer is inactive as a PfPKG inhibitor. We carried out crystallographic and molecular modeling studies to investigate the structural basis of its binding to PfPKG. We obtained a 2.54 Å atomic structure of PfPKG bound to (*R*)-RY-1-165.^29^ Inspection of the X-ray structure shows the inhibitor occupies the ATP-binding site of the N-lobe kinase domain, consistent with an ATP competitive mode of action. Contacts between the protein and the inhibitor are extensive throughout the ATP-binding pocket (Figure 1). The “DFG” (Asp–Phe–Gly) activation loop displays an “in” conformation although the D682 carboxylate atoms are facing away from the hydrophobic phenyl ring of the inhibitor.^33^ The phenyl ring sits in a hydrophobic pocket adjacent to the T618 gatekeeper residue 3.5 Å distal to the c-beta-methyl group of the threonine side chain. The structurally similar compound, RY-1-185, differs from RY-1-165 by the addition of a chloro group onto the phenyl ring adjacent to the gatekeeper residue. Furthermore, this chloro atom likely projects into the hydrophobic pocket (Figure S1). For simplicity of presentation, we have selected to show only RY-1-165 in models. A biaryl pyrimidine-diazole core, common to RY-1-185 and RY-1-165, occupies the adenine pocket of the ATP-binding site. It mimics hydrogen bonding interactions between the adenine-N3 and the polypeptide backbone of the “hinge” region of the inhibitor binding site (Figure 1A).^34^ The thiazole end of the molecule projects outward toward the C-terminus of the protein (C-tail) and displaces residues 811–819, which are unresolved in the structure, compared to a previously reported AMPPNP bound-PfPKG structure (PDB: 5DZC,^34^) (Figure 1B). The stereocenter of (*R*)-RY-1-165 attaches the thiazole moiety to the main core of the molecule. Inspection of the thiazole region shows close interactions with residues G623 and G624, which form a tight turn facing the main chain nitrogens toward a carbonyl of (*R*)-RY-1-165 (Figure 2). Backbone atoms of G624 make a hydrogen bond with the ketone oxygen of the *R*-enantiomer. We propose that this contact is responsible for stereoselectivity of RY-1-165 and RY-1-185.

**Figure 1:**
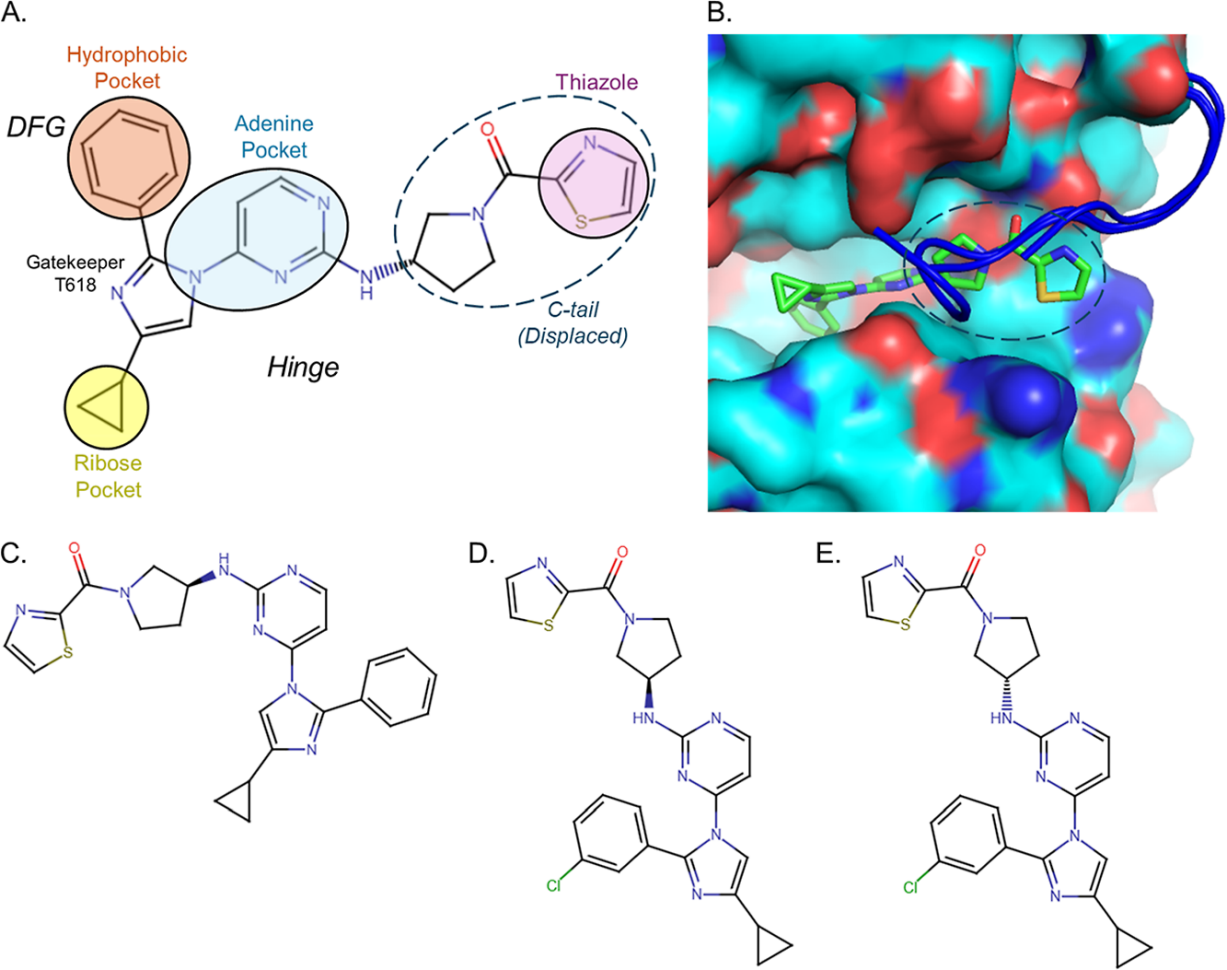
(*R*)-RY-1-165 displaces the c-terminal tail of PfPKG. (A) Schematic of (*R*)-RY-1-165 bound to PfPKG. (*R*)-RY-1-165 binds as a type 1 inhibitor in the ATP-binding site of the kinase. Its thiazole containing moiety (dashed circle) sits in the region of the binding site that is occupied by PfPKG’s c-terminal tail. The inhibitor’s contacts with PfPKG are indicated by solid circles and colored according to regions of the ATP binding pocket: ribose pocket (yellow), adenine pocket (blue), and hydrophobic pocket (orange). (B) Electrostatic surface potential of the 8EM8 binding pocket created in PyMOL (blue and red represent positively- and negatively charged surface regions, respectively). Superposition of other compound-bound PfPKG structures, 5DZC and 5EZR (blue coil), show c-terminal tails clashing with the thiazole containing moiety (dashed circle) of (*R*)-RY-1-165 (green). (C) (*S*)-RY-1-165 is inactive as a PfPKG inhibitor. (D) The chloro atom in (*R*)-RY-1-185 likely distends into the hydrophobic pocket. (E) The chloro atom in (*S*)-RY-1-185 projects into the gatekeeper residue, unlike its enantiomer, (*R*)-RY-1-185.

**Figure 2:**
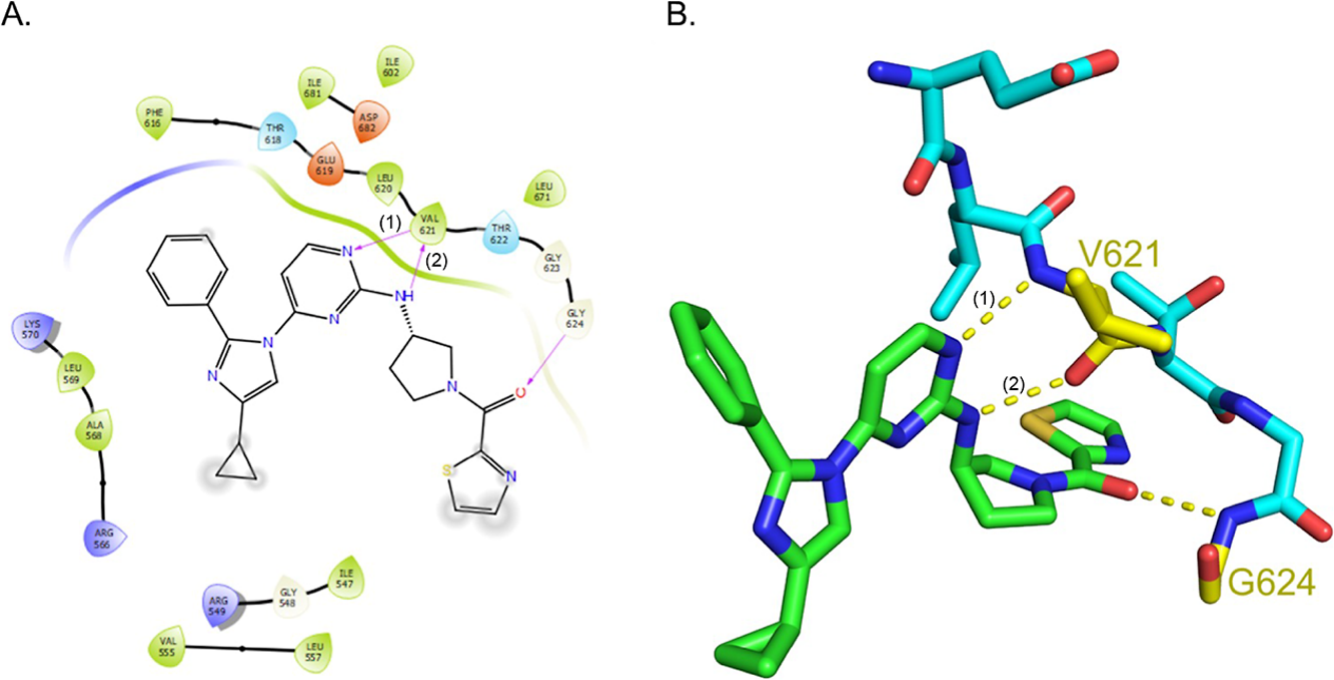
(A) Protein residues of PfPKG within 4 Å of (*R*)-RY-1-165 calculated with Maestro (Schrödinger, Inc.) (blue: cation; red: anion; green: hydrophobic). Close contacts of the molecule are shaded, and hydrogen bonds are indicated by arrows. (*R*)-RY-1-165 forms two hydrogen bonds with main-chain carbonyl oxygen and amide nitrogen of amino acid V621. The first hydrogen bond (1) forms between the partial positive charge of the hydrogen atom and the partial negative carbonyl oxygen of V261, creating a favorable electrostatic interaction. The second hydrogen bond (2) forms between another hydrogen atom from (*R*)-RY-1-165 and the amide nitrogen from V621. The hydrogen is attracted to the lone pair of electrons on the nitrogen atom, allowing for the formation of a stable hydrogen bond. V621 main chain atoms form hydrogen bonds in the “hinge” region of the adenine binding pocket. Backbone atoms of G624 make a hydrogen bond with the ketone oxygen of the *R-*enantiomer but not the *S*-enantiomer. (B) Interactions of (*R*)-RY-1-165 (green) with the peptide backbone of PfPKG (turquoise) visualized in PyMOL. Yellow lines indicate polar bond contacts.

### Larger Residue at the Gatekeeper Position in PfPKG Blocks Inhibition by (*R*)-RY-1-165 and (*R*)-RY-1-185 In Vitro

A key strategy for generating selectivity between parasite and human kinase inhibitors is to access a hydrophobic pocket, which in human kinases is blocked by larger residues such as methionine. The X-ray structure of (*R*)-RY-1-165 bound to PfPKG revealed that the molecule binds close to the “gatekeeper” residue of PfPKG, Thr_618_ (Figure 3). The phenyl moiety at the R2 position of the trisubstituted imidazole scaffold sits against the Thr_618_ methyl-group at a distance of 3.5 Å, extending into the hydrophobic pocket. To determine the effect of the gatekeeper residue on (*R*)-RY-1-165’s inhibition of the enzyme, we determined its IC_50_ against mutant PfPKG in which the Thr_618_ is replaced by glutamine (T618Q), an amino acid with a longer side-chain. This replacement resulted in a greater than 50-fold increase in the compound’s IC_50_ (Table 1, Figure S2), demonstrating that a residue with a short side chain is preferred at the “gatekeeper” position for the compound to access its binding pocket. Based on a comparative model, the chloro group on the phenyl ring of (*R*)-RY-1-185 should insert further into the hydrophobic pocket (Figure S1).

**Figure 3:**
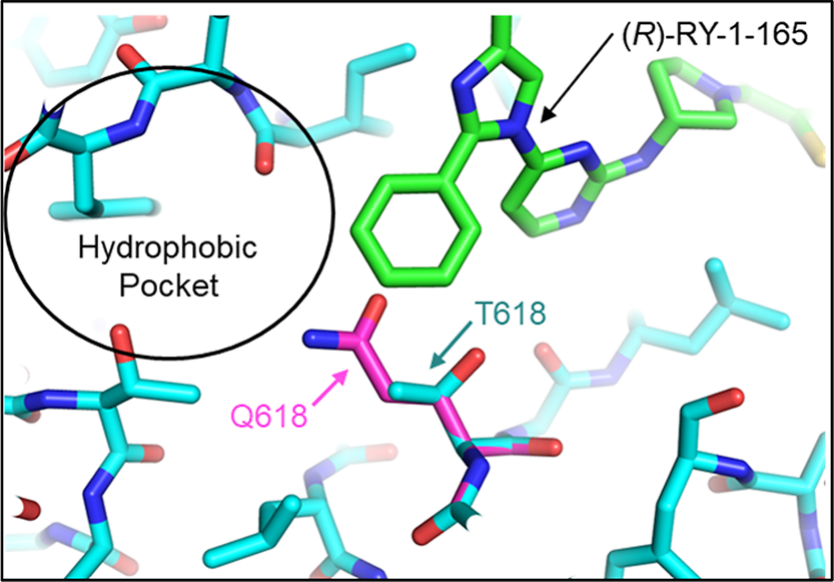
Structural model of (*R*)-RY-1-165 (green) bound to WT (turquoise) and PfPKG T618Q (pink) illustrates the binding site adjacent to the hydrophobic pocket, access to which is impacted by the identity of the gatekeeper residue. Structures were generated using PyMOL.

**Table 1:** Potency (IC_50_) and Activity (EC_50_) of (*R*)-RY-1-165 and (*R*)-RY-1-185 Against Selected recombinant enzymes and Parasites^a^

	enzyme IC_50_ (±SD)				P. falciparum EC_50_ (±SD)	
	PfPKG		PfCDPK1	PfCDPK4	WT	T618Q
	WT	T618Q	WT	WT		
(*R*)-RY-1-165	0.24 μM (±0.16)	>10.0 μM	10.10 μM (±3.60)	1.70 μM (±0.43)	11.00 μM (±1.10)	13.40 μM (±1.40)
(*R*)-RY-1-185	0.19 μM (±0.13)	>10.0 μM	6.69 μM (±2.25)	2.21 μM (±0.46)	6.50 μM (±0.42)	10.14 μM (±0.54)

a IC_50_ was determined using the ADP-Glo assay to quantify enzymatic activity (mean ± standard deviation, *n* = 2–3 experiments). EC_50_ was determined using PfPKG 3D7 (WT) or T618Q asexual stage parasites over a 72 h period (mean ± standard deviation, *n* = 1–2).

### (*R*)-RY-1-165 and (*R*)-RY-1-185 Have Multiple Cellular Targets

Next, to determine the specificity of (*R*)-RY-1-165 and (*R*)-RY-1-185 we tested the extent to which their antiparasitic activity is mediated via PfPKG inhibition. We established dose response curves of the two compounds for asexual stage parasites of wild type (3D7) and mutants expressing PfPKG T618Q. Since PfPKG enzyme carrying a T618Q mutation is recalcitrant to inhibition by (*R*)-RY-1-165 and (*R*)-RY-1-185 compared to WT enzyme (Table 1), parasites expressing it should be significantly less sensitive to inhibition by the compounds than 3D7. We found that the EC_50_ of the compounds in T618Q-expressing parasites was only marginally higher than against 3D7 (Table 1). This small difference contrasts with increases of 5 to 20-fold in EC_50_ of other PfPKG inhibitors in the same assay.^32^ These data demonstrate that the cellular activities of (*R*)-RY-1-165 and (*R*)-RY-1-185 are not driven solely by inhibition of PfPKG and imply the presence of additional targets in .

### Structure-Based Signature Motif Identifies Potential Kinases Which May Bind (*R*)-RY-1-165

To investigate if antiparasitic effects of (*R*)-RY-1-165 and (*R*)-RY-1-185 are caused by inhibition of kinases besides PfPKG, we examined sequence alignments of the 97 kinases encoded in the genome.^35^ We reasoned that any kinase inhibited by (*R*)-RY-1-165 should have a small gatekeeper residue (Thr, Ser, Cys, Gly or Ala) since a larger residue at this position disrupted PfPKG inhibition. Another structural determinant of PfPKG which drives binding of (*R*)-RY-1-165 is a glycine–glycine (G–G) turn (PfPKG 623–624) which contacts the ketone moiety of the thiazole *R*-stereoisomer and is predicted to be the primary driver of stereoselective activity of the molecules.^29,32^ These two glycines are four residues downstream from the gatekeeper residue and together, this 7-residue stretch of amino acids (PfPKG 618–624) makes vital contacts with (*R*)-RY-1-165 (Figure 2). We used a consensus sequence—“sXXXXGG” (s: T/S/C/G/A; X: any amino acid) to search 97 kinases. The search returned 4 kinases that matched the most conservative definition criteria of (i) a gatekeeper residue same or smaller than threonine and (ii) a G–G pair at positions homologous to 623 and 624 in PfPKG. These kinases were PfPKG (PF3D7_1436600.1), PfCDPK1 (PF3D7_0217500.1), PfCDPK4 (PF3D7_0717500.1), and a putative kinase STE/STE20 (PF3D7_0203100.1) (Figure 4). Intriguingly, PfCDPK1 and PfCDPK4 are known targets for drug discovery and promising candidates for potential off-target activity.

**Figure 4:**
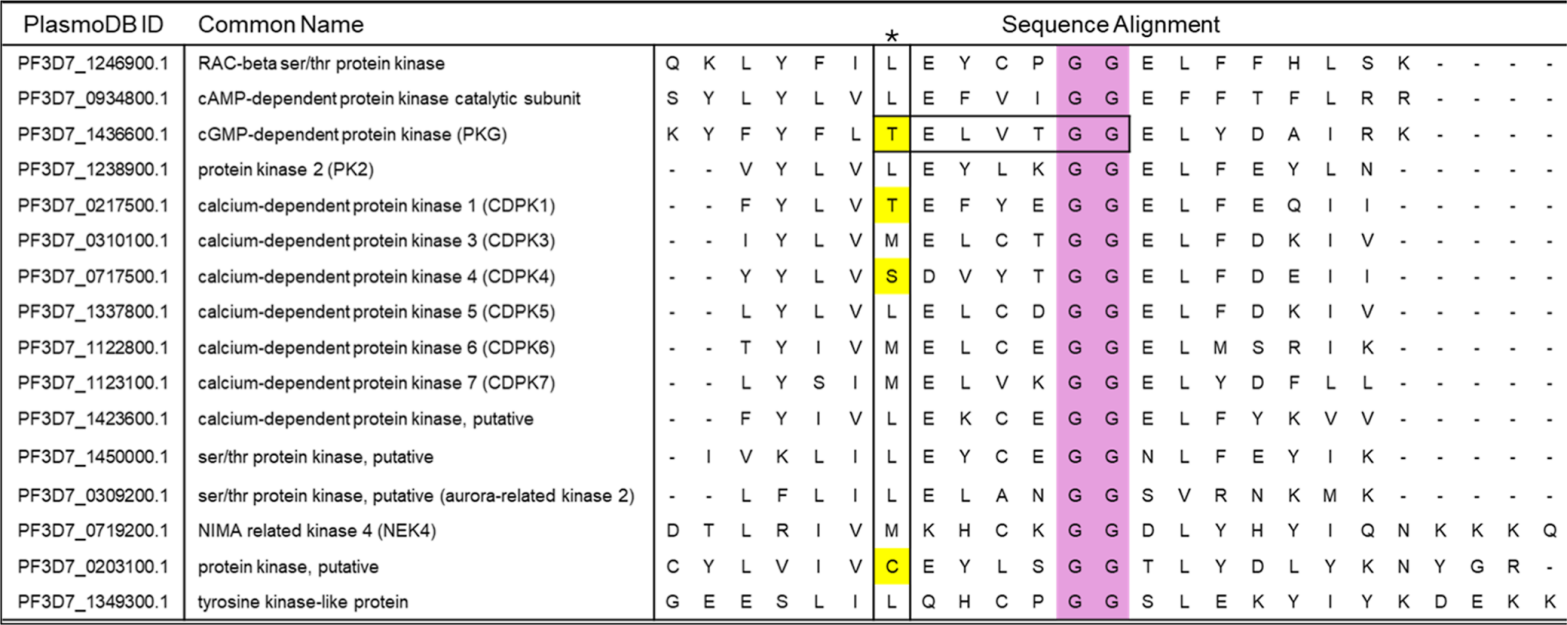
Focused sequence alignment of kinases based on conservative criteria. Sequence alignment of kinases most homologous to PfPKG with their gatekeeper residues marked by an asterisk. The four highlighted sequences represent kinases with both small gatekeeper residues (yellow) and the G–G pair at positions 5 and 6 (purple). The TELVTGG sequence of PfPKG is boxed.

### (*R*)-RY-1-165 and (*R*)-RY-1-185 Inhibit PfCDPK1 and PfCDPK4

To determine the potencies of (*R*)-RY-1-165 and (*R*)-RY-1-185 against PfCDPK1 and PfCDPK4, we established dose response curves using recombinant PfCDPK1 and PfCDPK4. PfCDPK4 was sensitive to (*R*)-RY-1-165 with an IC_50_ of 1.7 μM and to (*R*)-RY-1-185 with an IC_50_ of 2.21 μM (Table 1). Both compounds had a higher IC_50_ against PfCDPK1—10.1 and 6.69 μM, respectively (Table 1). To better understand the relative potencies against the two enzymes, we created models of (*R*)-RY-1-165 bound to PfCDPK1 and PfCDPK4, using known crystal structures of ligand bound proteins.

We used ROSETTA^36^ to create a structural model of PfCDPK1 based on the X-ray structure of CDPK1 (PDB: 3Q5I). We then superimposed the PfCDPK1 model and the (*R*)-RY-1-165-bound PfPKG X-ray structure (PDB 8EM8) using COOT^37^ and inspected the distal end of the ligand-binding pocket near the G–G pair. The overall structure of the peptide backbone near the G–G pair is highly similar between the two structures, suggesting the ability of PfCDPK1 to bind to the stereoisomer, (*R*)-RY-1-165 (Figure 5A). However, the side chain of the neighboring residue, Phe_146_ protrudes beneath the thiazole group of (*R*)-RY-1-165, potentially creating steric interference to binding. Residue Phe_146_ of PfCDPK1 is the structural equivalent of Leu_620_ in PfPKG and Val_149_ of PfCDPK4 and is the third residue of the “sXXXXGG” motif that points toward the bound compound. Some rotamer conformations of the larger Phe side chain can clash into the thiazole group, lowering the binding affinity of (*R*)-RY-1-165 for PfCDPK1 compared to PfCDPK4 which has a smaller Val (Figure 5B). (*R*)-RY-1-165’s higher IC_50_ against PfCDPK1 compared to PfCDPK4 is consistent with these structural models.

**Figure 5:**
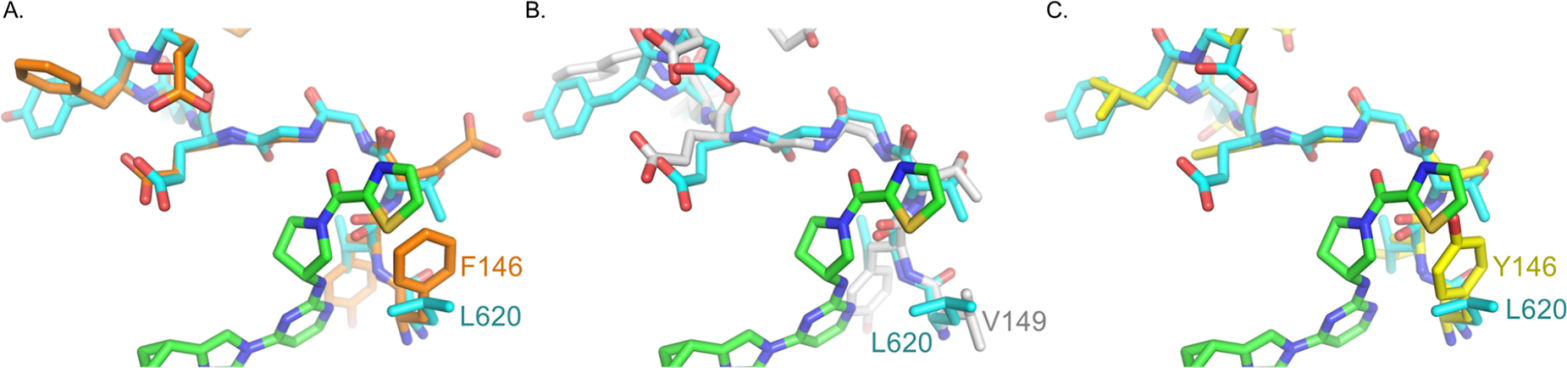
Predicted models of (*R*)-RY-1-165 bound to PfCDPK1, PfCDPK4 and STE/STE20, with PfPKG superimposed (turquoise). The size of the amino acid at the position equivalent to L_620_ in PfPKG affects (*R*)-RY-1-165 binding to PfCDPK1, PfCDPK4 and STE/STE20. (A) Predicted binding of (*R*)-RY-1-165 to PfCDPK1 shows that (*R*)-RY-1-165 fits into the ligand binding pocket but residue F_146_ clashes with the thiazole group. (B) Predicted binding of (*R*)-RY-1-165 to PfCDPK4 shows the conformation of the GG-elbow matches the structure of PfPKG when superimposed. (C) Predicted binding of (*R*)-RY-1-165 to STE/STE20, whose Y_146_ residue clashes with the thiazole group.

The fourth kinase containing the “sXXXXGG” motif—STE/STE20 (PF3D7_0203100.1) contains a large mobile amino acid, Tyr_146_ in the third position of the motif, similar to Phe_146_ of PfCDPK1. We created a model of its kinase domain to compare to the ligand bound structure (Figures 5C and S3). We predict that, like PfCDPK1, STE/STE20 will be relatively less sensitive to inhibition by (*R*)-RY-1-165 and (*R*)-RY-1-185.

Our data suggested that PfCDPK4 and to a lesser extent, PfCDPK1 and STE/STE20 could be additional targets of (*R*)-RY-1-165, and likely (*R*)-RY-1-185 in . While inhibition of PfCDPK4 is unlikely to impact asexual replication,^18^ blocking PfCDPK1 and STE/STE20 activities is expected to impair the asexual intraerythrocytic cycle since the individual loss of either enzyme attenuates the growth of asexual parasites.^17,38^

Since PfCDPK4 homologues in and are validated as drug targets in these parasites, we next tested the potency of both compounds against TgCDPK1 and CpCDPK1. Compared to PfCDPK4, CpCDPK1 and TgCDPK1 are significantly more sensitive to (*R*)-RY-1-165 and (*R*)-RY-1-185. The IC_50_ of both compounds for TgCDPK1 (Table 2, Figure S4) is over 30-fold lower than for PfCDPK4 (Table 1). Both CpCDPK1 and TgCDPK1 have Gly gatekeeper residues, which fully open the hydrophobic pocket for binding to the phenyl- and chlorophenyl-groups of (*R*)-RY-1-165 and (*R*)-RY-1-185, respectively. The Gly gatekeeper residues likely account for the high potency of the compounds against these enzymes. Consistent with this prediction, the TgCDPK1 G128 M mutant enzyme is at least 500–600-fold less sensitive and the CpCDPK1 G152 M mutant enzyme is at least 175–300× less sensitive to (*R*)-RY-1-165 and (*R*)-RY-1-185, respectively (Table 2).

**Table 2:** Potency (IC_50_) of (*R*)-RY-1-165 and (*R*)-RY-1-185 against TgCDPK1 and CpCDPK1 Enzyme**s** ^a^

TgCDPK1 IC_50_ (±SD)			CpCDPK1 IC_50_(±SD)		
	WT	G128M		WT	G152M
(*R*)-RY-1-165	0.05 μM (±0.02)	>30.0 μM	(*R*)-RY-1-165	0.17 μM (±0.05)	>30.0 μM
(*R*)-RY-1-185	0.06 μM (±0.02)	>30.0 μM	(*R*)-RY-1-185	0.09 μM (±0.03)	>30.0 μM

a IC_50_ were determined using the Kinase-Glo assay to quantify enzymatic activity (mean ± standard deviation, *n* = 2–3 experiments).

### (*R*)-RY-1-165 and (*R*)-RY-1-185 Inhibit and 

We tested the activity of (*R*)-RY-1-165 and (*R*)-RY-1-185 against and in vitro. replication, measured in HCT-8 cells, was inhibited with an EC_50_ of 17.90 μM (±0.49) by (*R*)-RY-1-165 and 10.80 μM (±1.48) by (*R*)-RY-1-185 (Figure S5). replication was measured by lysis of fibroblast host cells or colorimetric measurement of a β-galactosidase transgene. Both compounds displayed submicromolar EC_50_s against tachyzoites demonstrating they are very effective in blocking replication (Table 3). The relative sensitivity of to (*R*)-RY-1-165 and (*R*)-RY-1-185 is consistent with the compounds’ relative potency against TgCDPK1. Next, we tested if their action against relies solely on inhibition of TgCDPK1. We generated dose response curves using mutants expressing the TgCDPK1 gatekeeper mutant, G128 M^39^ and its parental line as control. The EC_50_ for G128 M parasites of (*R*)-RY-1-165 was 1.7-fold higher and of (*R*)-RY-1-185 was 1.8-fold higher compared to the parental strain (Table 3, Figure S6). The small increases in EC_50_ values suggest that both compounds have targets additional to TgCDPK1 in .

**Table 3:** (*R*)-RY-1-165 and (*R*)-RY-1-185 Have Multiple Targets in ^a^

T. gondii EC_50_ (±SD)								
	CONTROL	PKGT761Q	CONTROL	CDPK1 G128M	CONTROL	MAPKL1 L162Q	CONTROL	CDPK1 G128M; MAPKL1 L621Q
(*R*)-RY-1-165	0.26 μM (±0.16)	0.39 μM(±0.12)	0.86 μM (±0.14)	1.53 μM (±0.60)	0.53 μM (±0.15)	0.56 μM (±0.05)	0.54 μM (±0.0.02)	1.57 μM (±0.0.02)
(*R*)-RY-1-185	0.10 μM (±0.04)	0.17 μM (±0.16)	0.15 μM (±0.27)	0.30 μM (±0.27)	0.12 μM (±0.02)	0.33 μM (±0.0.02)	0.12 μM (±0.0.02)	1.21 μM (±0.0.02)

a Compound activity (EC_50_) against tachyzoites expressing mutants of TgPKG (T761Q), TgCDPK1 (G128M) and TgMAPKL-1 (L162Q) enzymes (mean ± standard deviation, *n* = 3–5 experiments each with 4 technical replicates).

We investigated PKG (TgPKG) and MAPKL-1 (TgMAPKL-1) as additional targets in . We reasoned that TgPKG could be a target since its kinase domain is over 70% identical to PfPKG, including conservation of the “TELVTGG” motif with Thr_761_ at the gatekeeper position. We were unable to test its inhibition directly since attempts to express recombinant TgPKG were unsuccessful. In lieu of biochemical assays, we utilized modeling to study interactions of TgPKG with (*R*)-RY-1-165 and (*R*)-RY-1-185. Our modeling predicts that both compounds bind to TgPKG in a manner similar to PfPKG and implies robust inhibition of the enzyme (Figure S7). To test if inhibition of TgPKG contributes to the antiparasitic activities of (*R*)-RY-1-165 and (*R*)-RY-1-185, we determined dose–response curves against WT and mutants expressing a Thr_761_ substitution (T761Q)^12^ (Table 3, Figure S8). (*R*)-RY-1-185 displayed an average EC_50_ of 94 nM against parental parasites and of 161 nM against the T761Q mutants, a 1.7-fold increase that is consistent with TgPKG being a target of the compound in . The results with (*R*)-RY-1-165 were mixed. In 3 experiments there was a 1.8–2.8-fold increase in EC_50_ against T761Q parasites relative to parental controls while in 2 experiments there was no change in the relative EC_50_.

Next, we investigated TgMAPKL-1 because it has a small gatekeeper residue (Ser_191_) and its genetic^40^ or pharmacological inhibition blocks parasite growth.^41–43^ Interestingly, several chemical scaffolds targeting TgCDPK1 also inhibit TgMAPKL-1.^41–43^ To test the contribution of TgMAPKL-1 inhibition to the cellular activities of (*R*)-RY-1-165 and (*R*)-RY-1-185, we utilized parasites carrying mutations in either only TgMAPKL-1 (TgMAPKL-1 L162Q) or both TgCDPK1 and TgMAPKL-1 (TgCDPK1 G128M; TgMAPKL-1 L162Q).^44^ (*R*)-RY-1-185 demonstrated an approximately 2.75-fold increase in EC_50_ against TgMAPKL-1 L162Q parasites (Table 3, Figure S9) and a 9-fold increase in EC_50_ against the double mutant TgCDPK1 G128M; TgMAPKL-1 L162Q (Table 3, Figure S10). This data show that TgCDPK1 and TgMAPKL-1 are targets of (*R*)-RY-1-185 in tachyzoites. The presence of multiple targets diminishes the degree of resistance caused from mutations in only one of the targets.

In contrast to (*R*)-RY-1-185, (*R*)-RY-1-165 did not demonstrate a significant change in EC_50_ against TgMAPKL-1 L162Q compared to the parental strain (Table 3, Figure S10). Its EC_50_ against the double mutant TgCDPK1 G128M; TgMAPKL-1 L162Q parasites was approximately 2.6-fold higher than the parental control. The similarity in the fold-changes of EC_50_ against the double mutant parasites and the TgCDPK1 G128 M mutant (1.7-fold) suggests that TgMAPKL-1 is unlikely to be a major target of (*R*)-RY-1-165. As in asexual stages, (*R*)-RY-1-165 appears to have multiple molecular targets in tachyzoites.

## Discussion

The kinome of apicomplexan parasites is an attractive source of potential drug targets since kinases are druggable and parasite enzymes have exploitable differences from their human counterparts. Several kinases from these parasites are amenable to specific and selective pharmacological inhibition.^11^ A compound targeting PI4K, MMV390048 has recently entered clinical trials.^11^ At least 50 kinases^45–47^ and 23 kinases^48^ are essential for the viability of at least one parasite stage. The genome is predicted to encode about 70 kinases.^49^ While their genetic validation is in its infancy, one kinase, CpCDPK1, is already known to be essential for parasite survival.^20^ X-ray crystallographic information is available for at least 12 kinases each from and and 1 from , providing impetus for structure-guided drug design of inhibitors.

Our results show that (*R*)-RY-1 series is effective against multiple kinases, PfPKG, PfCDPK1, and PfCDPK4. All three kinases are essential for parasite transmission to mosquitoes.^13,17,18,50^ Simultaneous inhibition of these kinases could have a synergistic effect on infection of the vector. (*R*)-RY-1-185 is more potent than (*R*)-RY-1-165 against all the tested kinases and parasites. The increase, although not large, is measurable. Our modeling suggests that (*R*)-RY-1-185’s higher potency derives from the chloro group’s insertion into the ‘hydrophobic pocket’ of these kinases. This may lead to a greater degree of hydrophobic interactions between the compound and the pocket. Ongoing medicinal chemistry to improve compound potency include exploration of the hydrophobic pocket occupied by the 3-chloro moiety. Substitutions on the pyrrolidine ring and the heterocyclic amide may also prove useful.

One target compound profile (TCP) for next generation of antimalarials described by the Medicines for Malaria Venture (MMV) is activity against gametocytes to block transmission (TCP 5).^51^ Molecules of the series could be developed to address this need. (*R*)-RY-1-185’s activity against asexual stages can be attributed to its inhibition of at least PfPKG and PfCDPK1. Additional targets in asexual blood stages mediating its activity could be kinases, such as STE/STE20, or other ATP-binding proteins. does not contain a MAPKL-1 homologue. We speculate that the relatively modest effect on asexual stages compared to tachyzoites may be due to the nonessential function of MAPK1 homologue in the asexual stages.^52^

Pathogen hopping has been used previously to leverage resources of antimalarial drug discovery to seed drug discovery efforts against neglected parasitic diseases.^53–55^ In our study, the (*R*)-RY-1 series displayed greatest potency against tachyzoites. Our results point to differential sensitivities of highly similar enzymes to the same molecule, existence of targets unique to and/or differences in the balance of activities carried out by the targeted kinases in the three parasites. They strongly suggest polypharmacology in the potent activity of the (*R*)-RY-1 series against tachyzoites. While poor expression of recombinant proteins prevented direct testing of TgPKG and TgMAPKL-1 enzymatic activities, the increased EC_50_ against parasites carrying one or more active site mutants, relative to wildtype, demonstrates that inhibition of these enzymes contributes to the antiparasitic effect of the compounds. Whereas TgPKG and TgCDPK1 contain the “sXXXXGG” sequence element that we predict is important for other parasite kinase targets, TgMAPKL-1 does not. The TgMAPKL-1 structure predicted by Alphafold contains several unstructured regions within the kinase domain that limit the accuracy of modeling inhibitor binding (UniProt Q5SC61). We posit that the solved structure of TgMAPKL-1 may reveal structural elements that recapitulate the interactions provided by the sequence element in other identified targets. Additional kinases with small gatekeeper residues, such as TGME49_239420 (A0A125YLH3_TOXGV) or TGME49_226540 (B9PMQ1_TOXGV), could also be targeted by these compounds. Unbiased approaches, such as chemoproteomics or selection of resistant parasites using (*R*)-RY-1 compounds, could be helpful in their identification.

Determining the effect of the (*R*)-RY-1 series on bradyzoites is an important avenue for additional exploration. Bradyzoites within tissue cysts persist in the host and are insensitive to standard therapies for acute toxoplasmosis. Circumstantial evidence implicates TgPKG, TgCDPK1 and TgMAPKL-1 in bradyzoite formation. Pharmacological inhibition of TgCDPK1, TgMAPKL-1 and TgPKG is associated with a upregulation of bradyzoite-associated markers^56–58^ and its deletion substantially decreases tissue cysts in infected mice.^40^ Therefore, blocking TgCDPK1, TgPKG and TgMAPKL-1 may inhibit both the lytic cycle and the formation of tissue cysts in vivo. Multikinase inhibitors are an attractive strategy for overcoming resistance as the parasite must develop resistance to the compound in more than one target. Indeed, polypharmacology is a hallmark of most human kinase inhibitors approved for treatment. Our results support an investigation of (*R*)-RY-1 analogs against in vivo.

## Methods

### Structural Studies

PyMOL software (Schrödinger, LLC.) was utilized for structure visualization and docking studies. Docking of (*R*)-RY-1-165 and (*S*)-RY-1-165 enantiomers was conducted using GLIDE (Schrödinger, Portland Oregon) using the structure of PfPKG bound to (*R*)-RY-1-165, PDB 8EM8. Superpositions of PfCDPK4 were conducted using COOT and PyMOL.

### PfPKG Enzyme Activity Assay

Direct inhibition of recombinant PfPKG phosphorylation activity was carried out using a previously described nonradioactive assay.^25^ The assay evaluates PfPKG activity based on changes in initial ATP concentration via luminescence after enzyme phosphorylation of peptide substrate PKCtide (ERMRPRKRQGSVRRRV) (SignalChem, Richmond, BC Canada). Enzyme kinase activity was measured in the presence of 26 μM PKCtide; 50.1 nM PfPKG; and 20 μM cGMP in a buffered solution containing 20 mM β-glycerol-phosphate, 20 mM MgCl_2_, 25 mM HEPES pH 7.5 (KOH), 0.1% BSA and 2 mM DTT.^25^ The reaction was activated by the addition of 10 μM ATP with incubation at 30 °C and 90 rpm agitation for 120 min. Assay to measure the level of PfPKG inhibition by Compound (*R*)-RY-1 series as well as determination of IC_50_ values was performed in a 3-fold serial dilution of each inhibitor starting at 12 μM. Each assay plate contained reaction wells with no enzyme, no PKCtide peptide substrate and no cGMP as internal controls.

### CDPK Enzyme Activity Assay

Recombinant expression of PfCDPK1, PfCDPK4, TgCDPK1 WT, TgCDPK1-G128M, CpCDPK1 and CpCDPK1-G152 M were purified as previously described.^14^ Protein kinase activity of the recombinant enzymes was assayed using 20 μM Synthide 2 peptide substrate: PLARTLSVAGLPGKK (American Peptide Company, Inc. Sunnyvale, CA); 8 nM PfCDPK1; 134 nM PfCDPK4; 3.1 nM TgCDPK1 WT; 20 nM TgCDPK1-G128M; 2.4 nM CpCDPK1; or 23.6 nM CpCDPK1-G152M. These reactions were performed in a buffer composed of 1 mM EGTA (pH 7.2), 10 mM MgCl_2_, 20 mM HEPES pH 7.5 (KOH), 0.1% BSA. The reactions were initiated with addition of 10 μM ATP and 2 mM CaCl_2_ as enzyme activation reagent. Reaction plates were incubated for 90 min with 90 rpm agitation at 30 °C. Inhibition concentration that gives 50% reduction in enzyme phosphorylation activity (IC_50_) was determined over curves with compound 3-fold serial dilutions from 30 μM (90 μM for PfCDPK1). KinaseGlo (Promega) luminescence-based assay in which luminescence is inversely related to kinase activity and directly related to ATP depletion was used as previously described.^14^

###  Growth Inhibition Assay

 lines were maintained with O+ human erythrocytes (BioIVT). WT 3D7A10 and T618Q parasites were kind gifts from Dr. David Fidock (Columbia University) and Dr. David Baker (London School of Tropical Medicine and Hygiene), respectively. Growth inhibition assays were performed as previously described.^32^ Parasites were grown in RPMI 1640 medium (Sigma-Aldrich, St. Louis, MO, U.S.A.) supplemented with 5 mg/mL Albumax (Gibco), 50 μg/mL hypoxanthine (Sigma-Aldrich), 2 mg/mL Na_2_CO_3_ (Sigma-Aldrich), 25 mM HEPES, and 50 μg/mL gentamicin (Gibco). The growth assay was initiated with cultures with >80% ring stage parasites, diluted to a parasitemia of 0.4% and a hematocrit of 1%. Parasites were transferred to a 96-well plate with test compounds, vehicle, or DHA as positive control, and maintained for 72 h in 5% CO_2_, 5% O_2_, and 90% N_2_ in a humidified chamber. Parasite viability at 72 h was determined by addition of SYBR Green I nucleic acid stain (Thermo Fisher Scientific, S7563) and Mitotracker Deep Red (Fisher M422426) followed by flow cytometry on a BD FACSVerse using BD FACSuite software. Data acquired were analyzed using Flowjo software. EC_50_ values were derived by nonlinear regression in Graph Pad Prism 7.

###  Growth Inhibition Assay

 strain UGA1 transfected to express nanoluciferase (Nluc) and obtained from the University of Arizona after propagation in newborn calves.^59^ HCT-8 cells were added to a 384-well plate and allowed to grow for 48 h to reach 75–90% confluence. The medium was removed, and test compounds were added in serial dilutions prior to the addition of 1000 oocysts per well in RPMI 1640 medium supplemented with 10% heat inactivated FBS and 1% penicillin streptomycin. Plates were incubated for 48 h; Nano-Glo luciferase reagent (Promega, Madison, WI) was then added, and the plates were read on an EnVision multilabel plate reader (PerkinElmer, Waltham, MA). EC_50_ curves were calculated as previously described using Prism v6.07 (GraphPad Software, La Jolla, CA).

### Host Cell and Culture

Human Foreskin Fibroblasts (HFF) were cultured in DMEM, with d-glucose (4.5 g/L), l-glutamine, and sodium pyruvate (110 mg/L) (Gibco) that were further supplemented with 10% Fetal Clone 1 serum (FCS)­(HyClone), 50 U/mL penicillin (Gibco), 50 μL/mL streptomycin (Gibco), and 1× GlutaMax (Gibco). HFF cells were cultured in T25 flasks (Corning) in a humidified incubator at 37 °C and 5% CO_2_. For culture, media was replaced with DMEM, as described above, with 1% FCS. Mutant strains were created as described in.^44^

###  Proliferation EC_50_s: *Tg*MAPKL1^162Q^ and Parental Strain

An RH strain of with an L162Q substitution in TgMAPKL1 introduced by CRISPR/Cas9 site directed mutagenesis into a clone expressing β-galactosidase was used to determine the EC_50_s for proliferation. Compounds were dissolved in DMSO and serially diluted 4-fold across the first 11 columns of a 96 well plate from the highest concentration of 25 μM leaving the final column with no drugs. was added to the first 11 columns of the 96 well plate at 4000 parasites per well. The last column contained no compound. Plates were incubated 72 h at 37 °C and 5% CO_2_. were quantified by spectrophotometrically measuring the β-galactosidase concentration of each well after cell lysis and exposure to chlorophenol red β-galactopyranose (Sigma-Aldrich, St. Louis, MO) substrate. Each compound was tested at least three times in quadruplicate.

###  Proliferation EC_50_s

Presence of desired mutations in parasite lines, *Tg*CDPK1^G128M^, *Tg*CDPK1^G128M^ + *Tg*MAPKL1^L162Q^ and *Tg*PKG^T671Q^ was verified through sequencing. Compounds were dissolved in DMSO and serially diluted 4-fold across the first 10 wells of a 96 well plate, with the highest drug concentration in the first column being 25 μM. The strain of was then added to the first 11 columns of the 96-well plate at a density of 4000 parasites per well. The 11th column contained no compounds, and the 12th column contained no parasites and no compound. After 5 days of incubation at 37 °C and 5% CO_2_, cells were washed with PBS and fixed with 4% PFA in PBS (Thermo Fisher Scientific, Waltham, MA, USA). After fixing, cells were washed three times with PBS and stained with 300 nM DAPI in PBS before being washed again three times with PBS.

Images were acquired in an ImageXpress Pico Automated Cell Imaging System (Molecular Devices, San Jose, CA, USA) with a 4× objective using UV fluorescence. Following imaging, ImageXpress software was used to count objects with a fluorescence intensity ≥8 and a size of 12–30 μm, giving a total count of host cell nuclei for each well. EC_50_ were determined based on the number of remaining fibroblast nuclei using a nonlinear regression analysis with GraphPad Prism software version 10.0.0 for Windows, GraphPad Software, Boston, Massachusetts USA. Each compound was tested in ≥3 experiments in quadruplicate rows.

## Supplementary Material



## Notes

Models of molecule binding to protein targets, dose response curves of molecules against target kinases and dose response curves of molecules against and ­(PDF)

## ○

UC San Diego, La Jolla CA, 92093, USA
