# Supplementary material for: Versatile Imidazole Scaffold with Potent Activity against Multiple Apicomplexan Parasites
Source: ACS Infect Dis. 2025 May 8;11(6):1497–507. doi: 10.1021/acsinfecdis.5c00049 (PMC12333026; doi:10.1021/acsinfecdis.5c00049)
Supplement: Supplementary file 1 [file id5c00049_si_001.pdf]

Supporting Information

A versatile imidazole scaffold with potent activity against multiple apicomplexan parasites

Monique Khim[1,2], Jemma Montgomery[3], Mariana Laureano De Souza[4#], Melvin Delvillar[4], Lyssa J. Weible[5], Mayuri Prabakaran[5], Matthew A. Hulverson[5], Tyler Eck[6], Rammohan Y. Bheemanabonia[6], P. Holland Alday[3,7], David P. Rotella[6], J. Stone Doggett[3,7], Bart L. Staker[1,2], Kayode K. Ojo[5,8], Purnima Bhanot[4*]

[1] Seattle Structural Genomics Center for Infectious Disease, Seattle, Washington, 98109, United States.

[2] Center for Global Infectious Disease Research, Seattle Children's Research Institute, Seattle, Washington, 98109, United States.

[3] Divisions of Infectious Diseases and Research, VA Portland Healthcare System, Portland, Oregon 97239, United States.

[4] Department of Microbiology, Biochemistry and Molecular Genetics, Rutgers New Jersey Medical School, Newark, New Jersey 07103, United States.

[5] Department of Medicine, Division of Allergy and Infectious Diseases, Center for Emerging and Reemerging Infectious Diseases, University of Washington, Seattle, Washington 98109, United States.

[6] Department of Chemistry and Biochemistry and Sokol Institute of Pharmaceutical Life Sciences, Montclair State University, Montclair, New Jersey 07043, United States.

[7] Division of Infectious Diseases, Oregon Health & Science University School of Medicine, Portland, Oregon 97239, United States.

[8] Department of Global Health, University of Washington, Seattle, Washington 98195, United States.

# Current address: UC San Diego, School of Medicine, Department of Pediatrics, La Jolla, California 92093, United States.

*To whom correspondence should be addressed: bhanotpu@njms.rutgers.edu

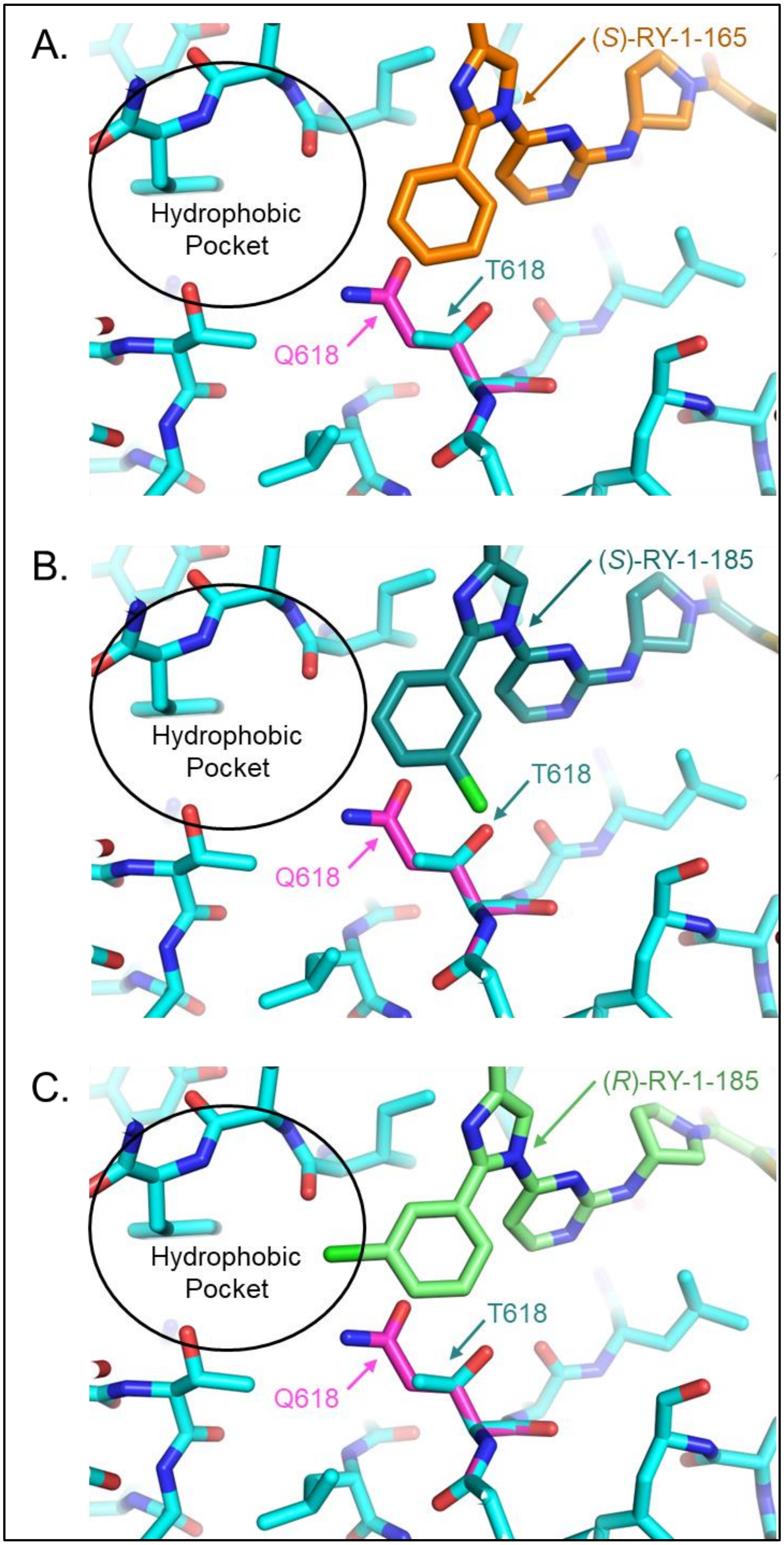

Figure S1. Modeling of (*S*)-RY-1-165 and (*S*)-RY-1-185 (teal) bound to WT PfPKG (T618) or gatekeeper mutant PfPKG (Q618). A. (*S*)-RY-1-165 (orange) bound to WT PfPKG (turquoise) or mutant PfPKG (pink). B. (*S*)-RY-1-185 (teal) bound to WT PfPKG (turquoise) or mutant PfPKG (pink). C. The chloro group on the phenyl ring of (*R*)-RY-1-185 (green) inserts further into the hydrophobic pocket of the enzyme.

A.

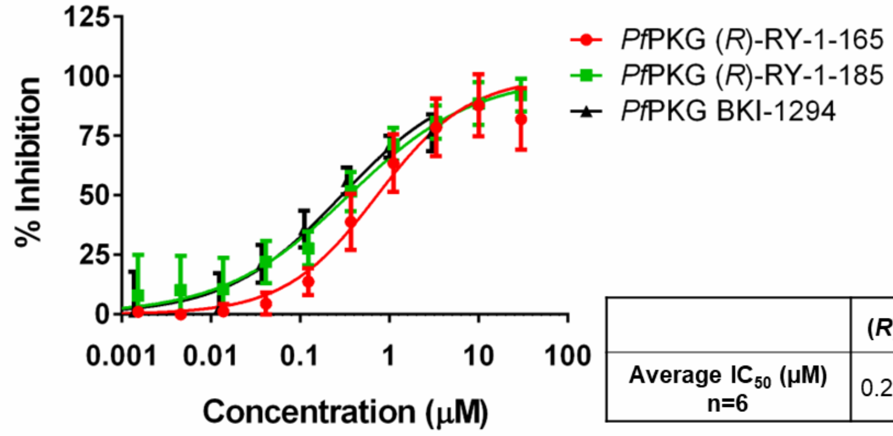

	**(*R*)-RY-1-165**	**(*R*)-RY-1-185**	**BKI-1294**
**Average IC$_{50}$ (μM)** **n=6**	0.244 (±0.161)	0.186 (±0.132)	0.167 (±0.144)

B.

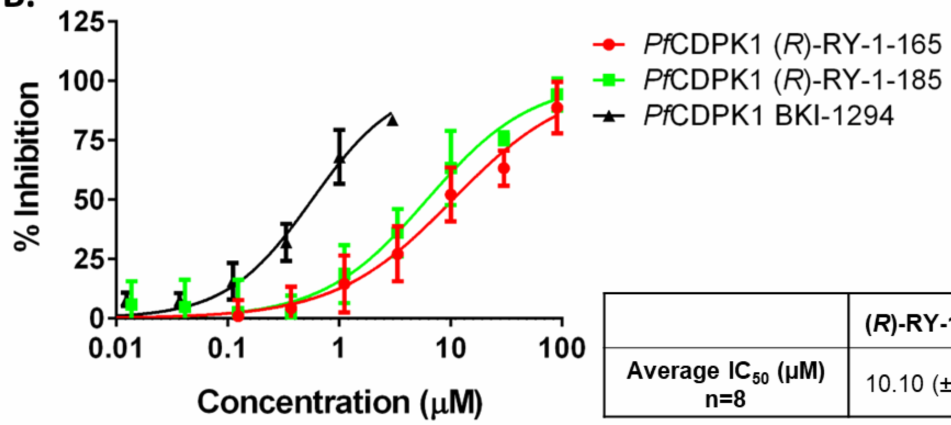

	**(*R*)-RY-1-165**	**(*R*)-RY-1-185**	**BKI-1294**
**Average IC$_{50}$ (μM)** **n=8**	10.10 (±3.60)	6.69 (±2.25)	0.59 (±0.18)

C.

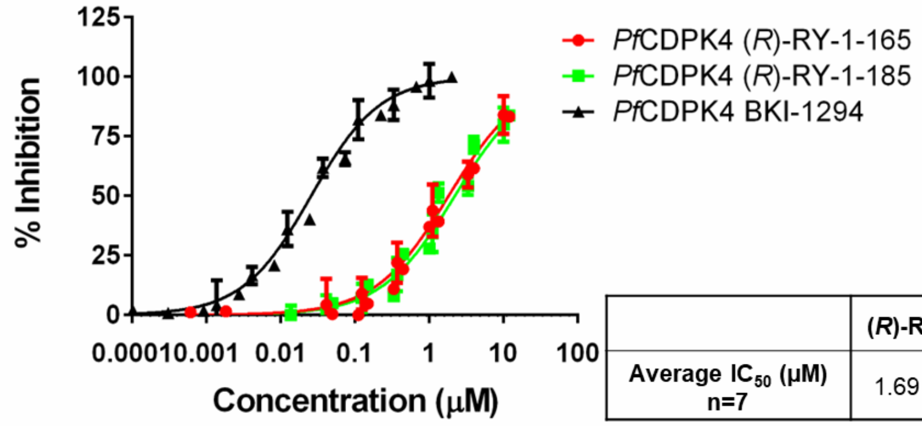

	**(*R*)-RY-1-165**	**(*R*)-RY-1-185**	**BKI-1294**
**Average IC$_{50}$ (μM)** **n=7**	1.69 (±0.43)	2.21 (±0.46)	0.027 (±0.007)

Figure S2. Dose-response curves against recombinant PfPKG (A), PfCDPK (B), and PfCDPK4 (C) enzymes. (*R*)-RY-1-165, (*R*)-RY-1-185 and BKI-1294 (a bumped kinase inhibitor of *C. parvum*) were tested against each enzyme. Data shown are mean ± standard deviation of ≥6 experiments.

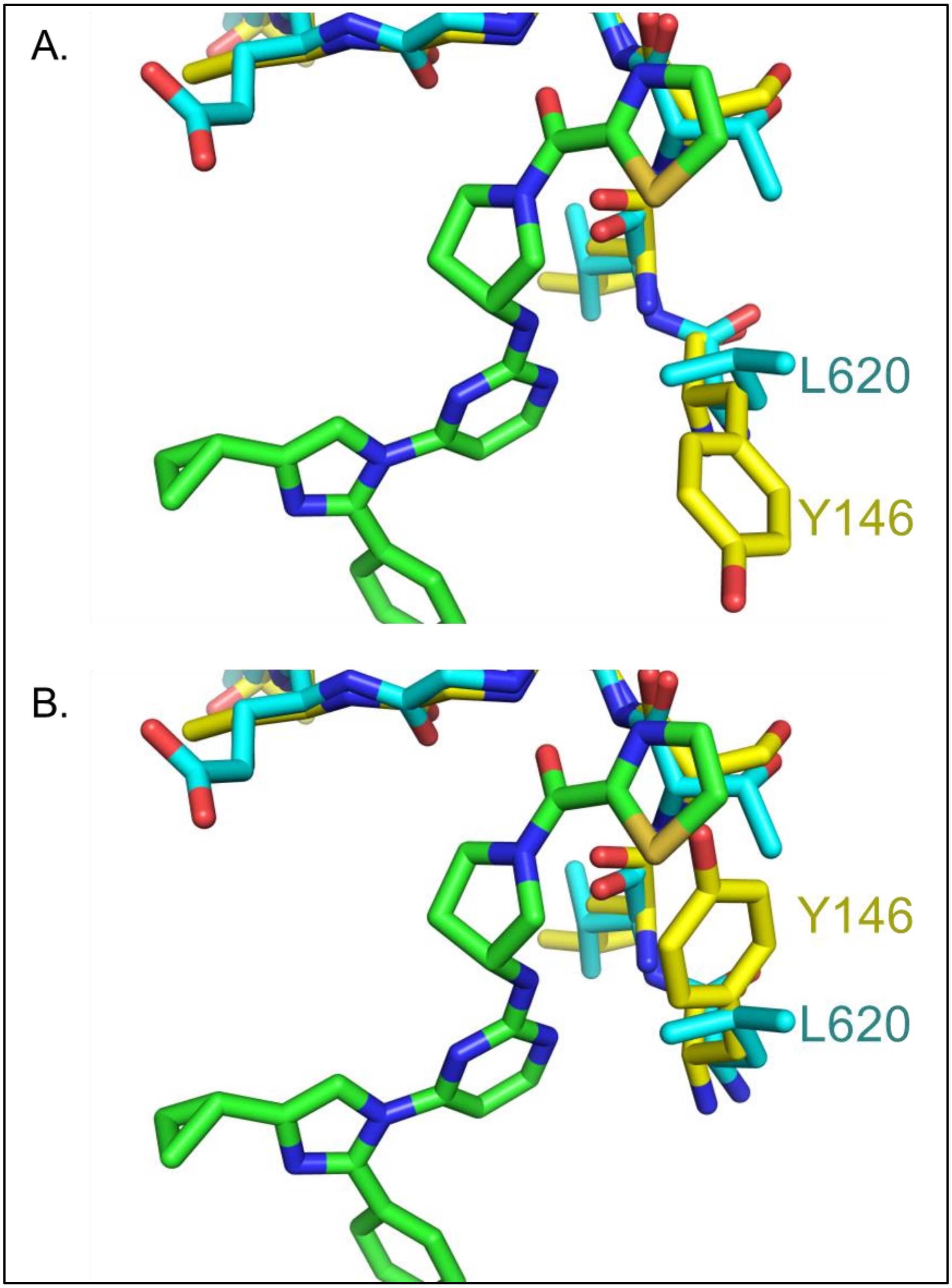

Figure S3. Predicted binding model of STE/STE20 (PF3D7_0203100.1) suggests two dominant rotamer positions for Y146. A. PyMOL predicts that the most common rotamer of Y146 (47%) does not collide with (*R*)-RY-1-165. B. The second-most predicted rotamer of Y146 (32.5%) is anticipated to clash with the thiazole group of (*R*)-RY-1-165.

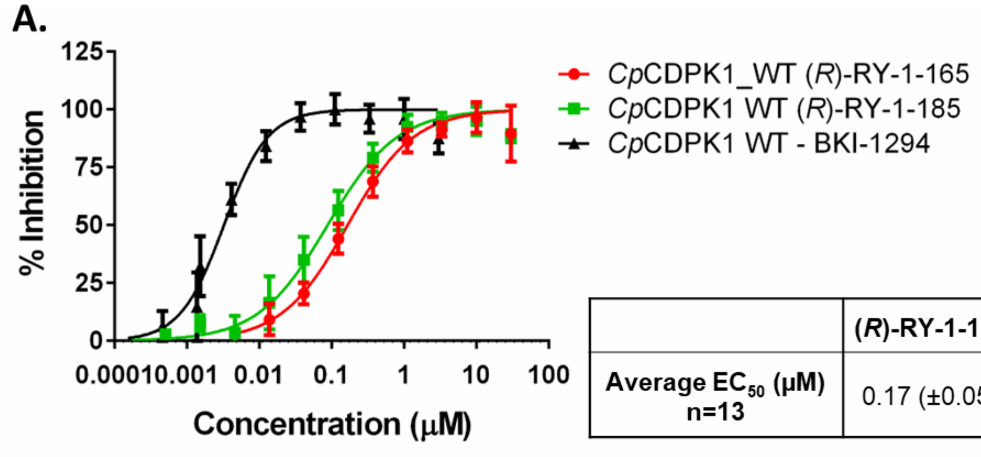

	(*R*)-RY-1-165	(*R*)-RY-1-185	BKI-1294
**Average EC_{50} (μM) n=13**	0.17 (±0.05)	0.09 (±0.04)	0.003 (±0.001)

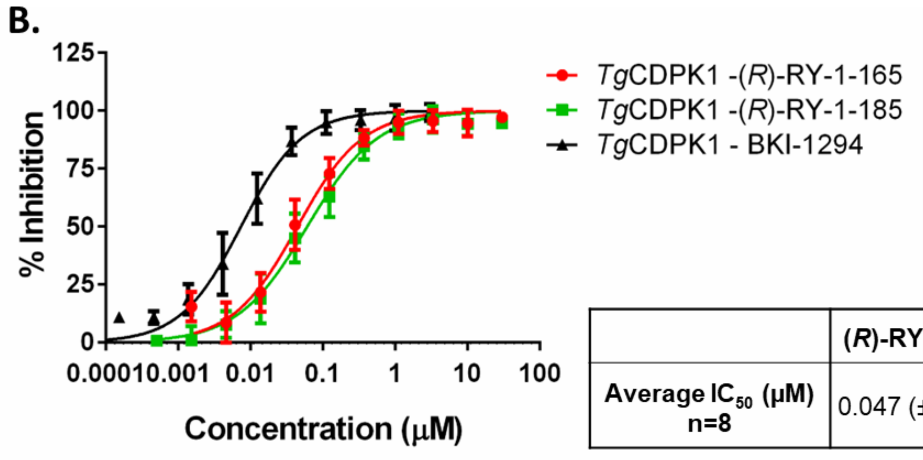

	(*R*)-RY-1-165	(*R*)-RY-1-185	BKI-1294
**Average IC_{50} (μM) n=8**	0.047 (±0.016)	0.065 (±0.022)	0.008 (±0.003)

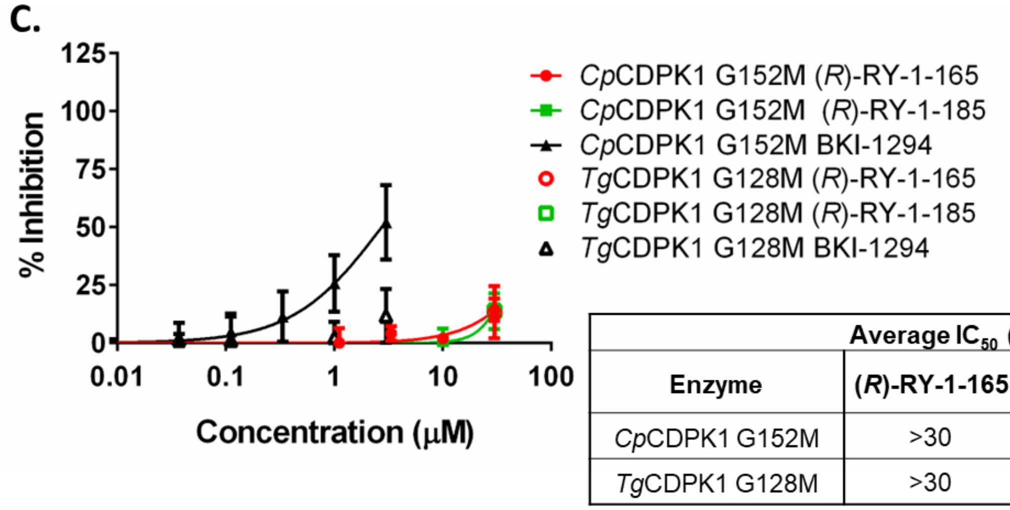

**Average IC_{50} (μM), n=2**			
**Enzyme**	**(*R*)-RY-1-165**	**(*R*)-RY-1-185**	**BKI-1294**
*Cp*CDPK1 G152M	>30	>30	>3
*Tg*CDPK1 G128M	>30	>30	>3

Figure S4. Dose-response curves against recombinant CpCDPK1 (A), TgCDPK1 (B), and their respective gatekeeper mutants (C). (*R*)-RY-1-165, (*R*)-RY-1-185 and BKI-1294 were tested against each enzyme. Data shown are mean ± standard deviation of ≥2 experiments

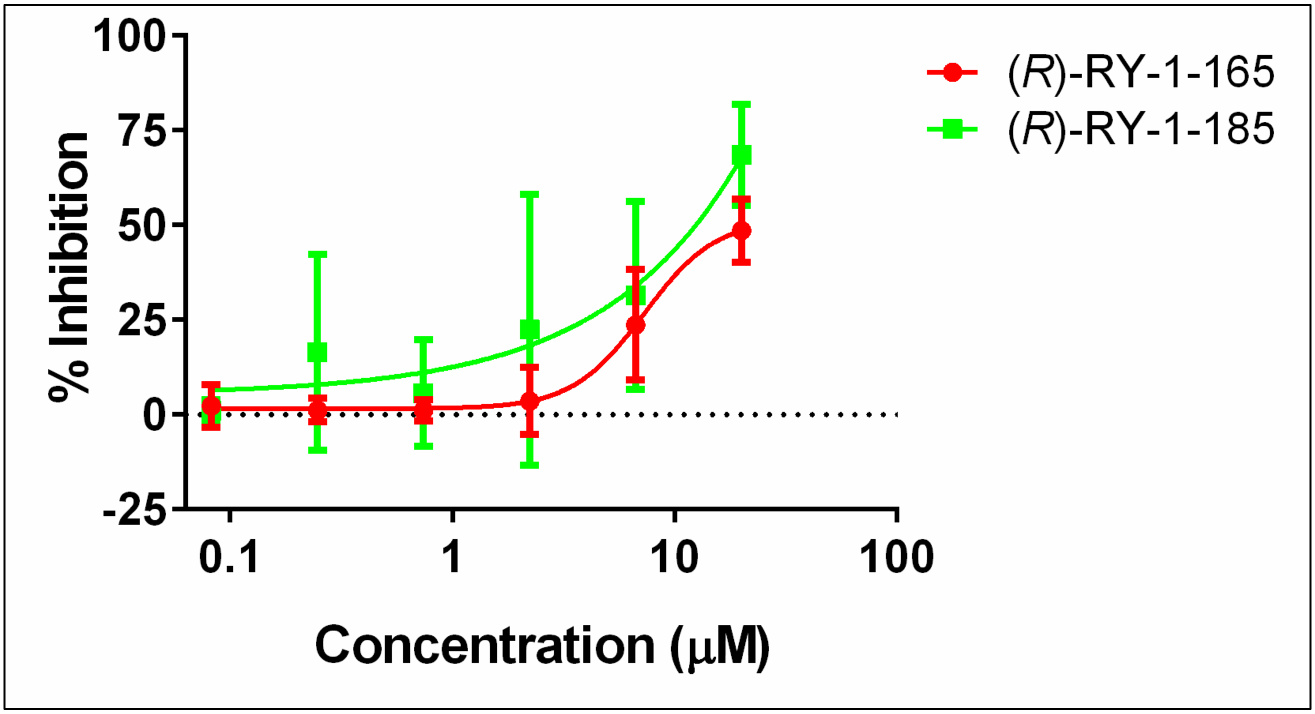

Figure S5. Dose-response curves against *C. parvum* trophozoite stage parasite of (*R*)-RY-1-165 (A) and (*R*)-RY-1-185 (B). Data shown are mean ± standard deviation of 2 experiments.

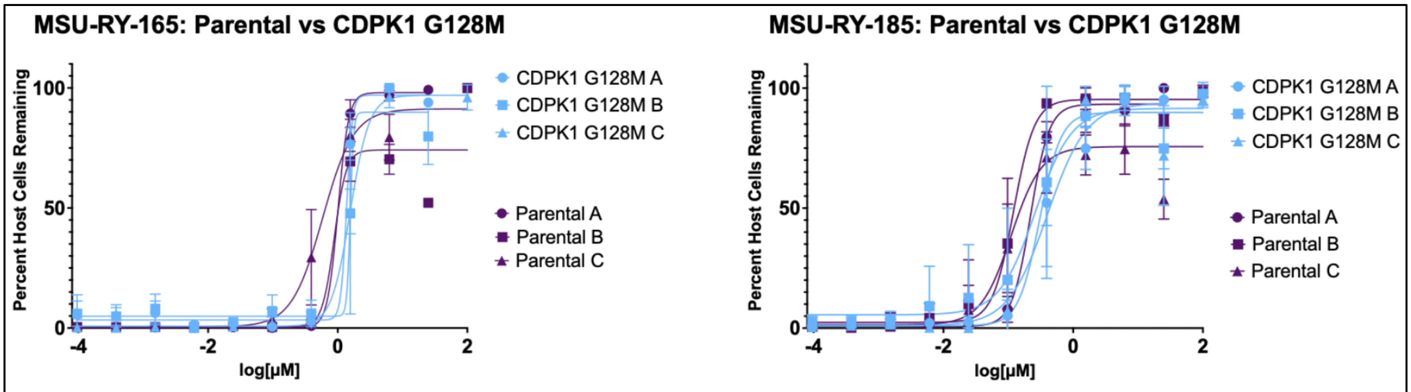

	**(*R*)-RY-1-165**	
	Parental	CDPK1 G128M
Trial A (μM)	1.009	1.369
Trial B (μM)	0.883	1.561
Trial C (μM)	0.585	1.566
Combined EC_{50} (μM)	0.857	1.527

	**(*R*)-RY-1-185**	
	Parental	CDPK1 G128M
Trial A (μM)	0.221	0.400
Trial B (μM)	0.121	0.255
Trial C (μM)	0.111	0.306
Combined EC_{50} (μM)	0.152	0.303

Figure S6. Dose-response curves against *T. gondii* parasites expressing either TgCDPK1 gatekeeper mutant enzyme or WT enzyme (parental). Data shown are mean (n=3) ± standard deviation.

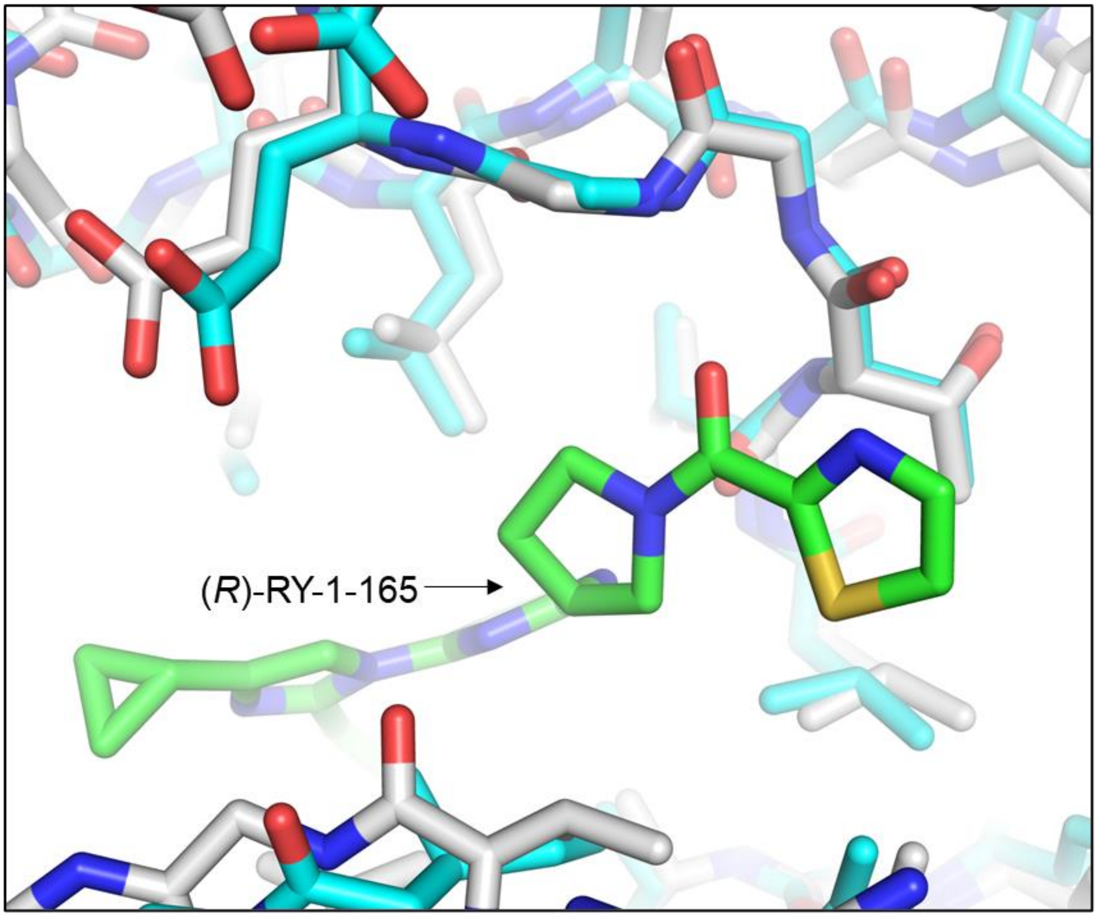

Figure S7. Predicted model for (*R*)-RY-1-165 (green) interaction with TgPKG. Superposition of PfPKG 3D7 (turquoise) with TgPKG (grey).

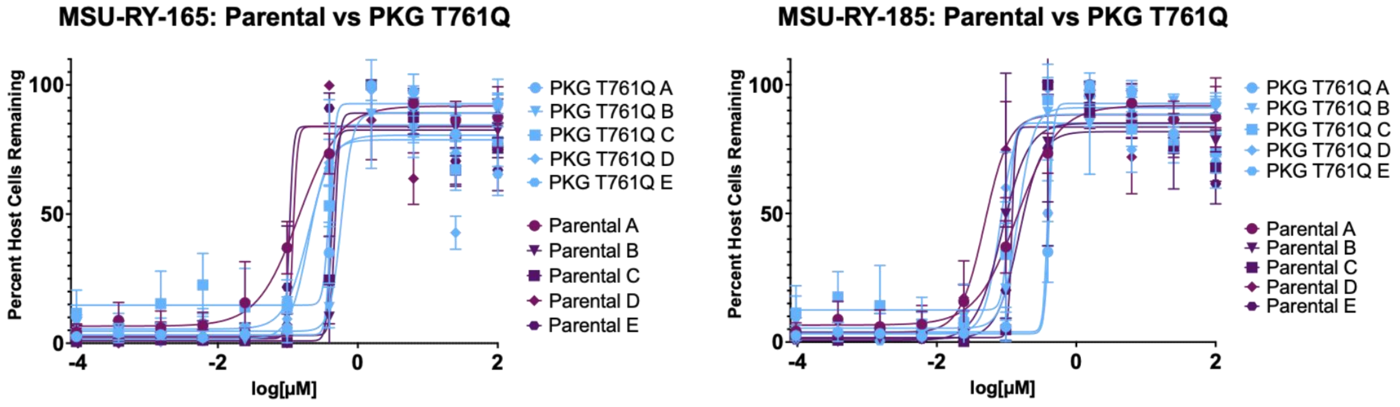

	(*R*)-RY-1-165	
	Parental	PKG T761Q
Trial A (µM)	0.144	0.409
Trial B (µM)	0.473	0.579
Trial C (µM)	0.426	0.384
Trial D (µM)	0.123	0.247
Trial E (µM)	0.102	0.198
Combined EC_{50} (µM)	0.260	0.390

	(*R*)-RY-1-185	
	Parental	PKG T761Q
Trial A (µM)	0.145	0.409
Trial B (µM)	0.083	0.146
Trial C (µM)	0.124	0.106
Trial D (µM)	0.049	0.086
Trial E (µM)	0.152	0.381
Combined EC_{50} (µM)	0.098	0.175

Figure S8. Dose-response curves of (*R*)-RY-1-165 and (*R*)-RY-1-185 against *T. gondii* parasites expressing either TgPKG gatekeeper mutant enzyme (T761Q) or WT enzyme (parental). Data shown are mean (n=3) ± standard deviation.

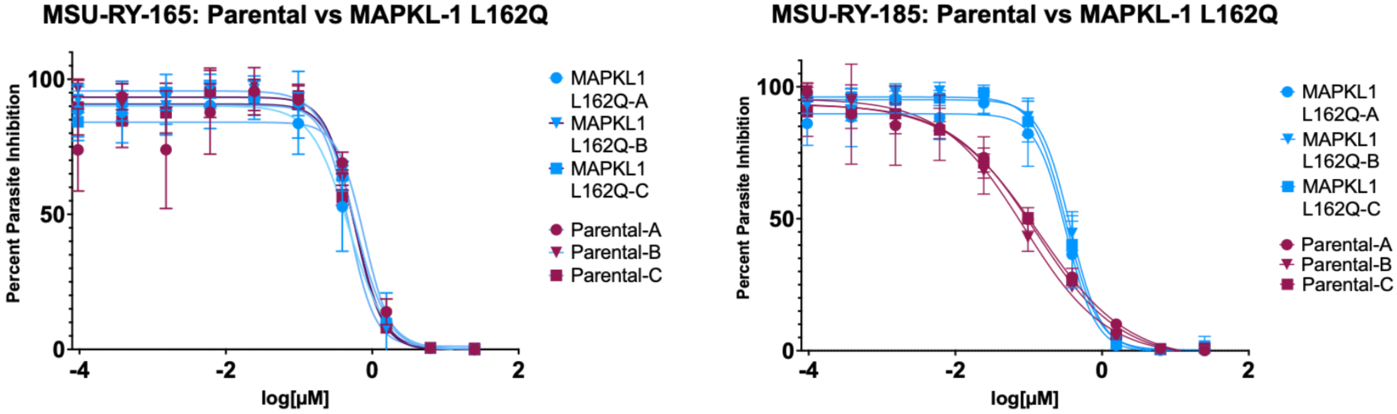

	(*R*)-RY-1-165	
	Parental	MAPKL-1 L162Q
Trial A (μM)	0.770	0.480
Trial B (μM)	0.566	0.567
Trial C (μM)	0.484	0.583
Combined EC_{50} (μM)	0.528	0.558

	(*R*)-RY-1-185	
	Parental	MAPKL-1 L162Q
Trial A (μM)	0.135	0.326
Trial B (μM)	0.087	0.366
Trial C (μM)	0.124	0.325
Combined EC_{50} (μM)	0.117	0.326

Figure S9. Dose-response curves of (*R*)-RY-1-165 and (*R*)-RY-1-185 against *T. gondii* parasites expressing either MAPKL-1 mutant (L162Q) or WT enzyme (parental).

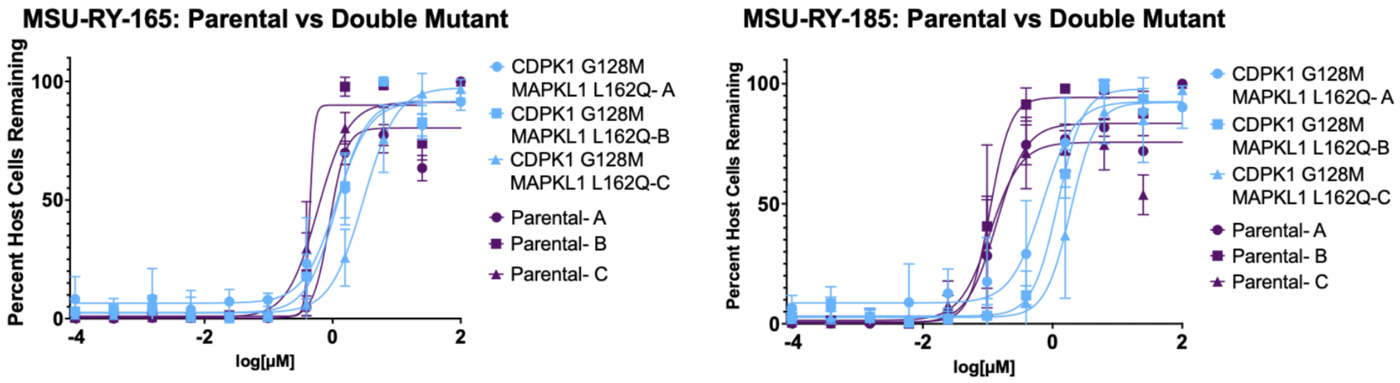

	**(*R*)-RY-1-165**	
	Parental	CDPK1 G128M MAPKL1 L126Q
Trial A (µM)	0.895	1.150
Trial B (µM)	0.440	1.157
Trial C (µM)	0.586	3.024
Combined EC_{50} (µM)	0.537	1.574

	**(*R*)-RY-1-185**	
	Parental	CDPK1 G128M MAPKL1 L126Q
Trial A (µM)	0.137	0.717
Trial B (µM)	0.109	1.223
Trial C (µM)	0.112	1.904
Combined EC_{50} (µM)	0.117	1.211

Figure S10. Dose-response curves of (*R*)-RY-1-165 and (*R*)-RY-1-185 against *T. gondii* parasites expressing either mutants of both TgCDPK1 and MAPKL-1 enzymes, or WT enzymes (parental).
